# DNA Alkylation Damage by Nitrosamines and Relevant DNA Repair Pathways

**DOI:** 10.3390/ijms24054684

**Published:** 2023-02-28

**Authors:** Jörg Fahrer, Markus Christmann

**Affiliations:** 1 Division of Food Chemistry and Toxicology, Department of Chemistry, RPTU Kaiserslautern-Landau, Erwin-Schrödinger Strasse 52, D-67663 Kaiserslautern, Germany; 2 Department of Toxicology, University Medical Center Mainz, Obere Zahlbacher Strasse 67, D-55131 Mainz, Germany

**Keywords:** *N*-nitroso compounds, *N*-nitrosamines, DNA alkylation, DNA damage, DNA repair, MGMT, AAG, ALKBH, BER, NER, TLS

## Abstract

Nitrosamines occur widespread in food, drinking water, cosmetics, as well as tobacco smoke and can arise endogenously. More recently, nitrosamines have been detected as impurities in various drugs. This is of particular concern as nitrosamines are alkylating agents that are genotoxic and carcinogenic. We first summarize the current knowledge on the different sources and chemical nature of alkylating agents with a focus on relevant nitrosamines. Subsequently, we present the major DNA alkylation adducts induced by nitrosamines upon their metabolic activation by CYP450 monooxygenases. We then describe the DNA repair pathways engaged by the various DNA alkylation adducts, which include base excision repair, direct damage reversal by MGMT and ALKBH, as well as nucleotide excision repair. Their roles in the protection against the genotoxic and carcinogenic effects of nitrosamines are highlighted. Finally, we address DNA translesion synthesis as a DNA damage tolerance mechanism relevant to DNA alkylation adducts.

## 1. Sources of DNA Alkylation Damage

**Figure 1:**
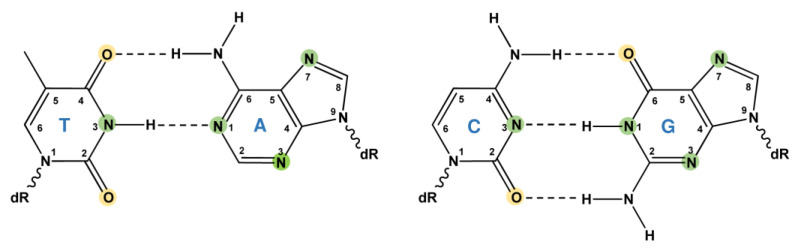
Alkylation of DNA bases. Alkylating agents of the S_N_1-type alkylate O- and N-atoms, whereas S_N_2 reagents mainly alkylate N-atoms. O-Atoms are depicted in yellow, whereas N-atoms are shown in green. dR: deoxyribose moiety.

DNA alkylation lesions are one of the most common types of DNA damage and can be induced by endogenous compounds, environmental agents, and alkylating drugs used in anticancer therapy. These alkylating agents are mutagenic, toxic, clastogenic, and teratogenic and represent well-known human carcinogens [1]. Depending on the given alkylating agent, different positions in the DNA can be attacked via nucleophilic substitution (S_N_-reaction). Alkylating agents can act via a S_N_1 or a S_N_2 reaction. During the S_N_2 (second-order nucleophilic substitution) reaction, the addition of the nucleophile and the elimination of the leaving group occur simultaneously. In opposition to that, during the S_N_1 (first-order nucleophilic substitution) reaction, the addition of the nucleophile and the elimination of the leaving group occur in two separated steps. The S_N_1 reaction is more important when the targeted carbon atom within the alkylating agent is surrounded by interfering bulky groups. Generally, alkylating agents can form adducts at all O- and N-atoms of purines and pyrimidines (Figure 1), as well as at phosphotriesters of the DNA backbone. While alkylating agents of the S_N_1-type alkylate O- and N-atoms, S_N_2 reagents mainly alkylate N-atoms [2]. 

*O*-alkylations are highly mutagenic and cytotoxic [3]. Although only amounting to less than 8% of total alkylations [2], *O*^6^-alkylguanine represents the most critical type of DNA alkylation damage. *O*^6^-methylguanine (*O*^6^-MeG) and *O*^6^-ethylguanine (*O*^6^-EtG) can mispair with thymine, leading to GC → AT transition mutations following two rounds of replication [4]. *O*^4^-methylthymine (*O*^4^-MeT) also represents a pre-mutagenic DNA lesion, but is only induced in minor amounts. *N*-alkylations are predominantly cytotoxic and less mutagenic, although recent in vivo data indicate mutagenicity for the replication-blocking lesion N3-methyladenine (N3-MeA) [5]. 

Endogenous alkylation (methylation) can occur by the intracellular methyl group donor S-adenosyl-l-methionine (SAM) [6] via nitrosation [7]. Whereas SAM acts by a S_N_2 mechanism and generates N7-Methylguanine (N7-MeG) and N3-MeA [6,8], enzymatic nitrosation of glycine and glycine derivatives as well as of bile acids predominantly forms *O*^6^-alkylating agents [7,9,10]. In addition, it is well established that bacteria in the stomach and gut catalyze the nitrosation of various secondary amines derived from ingested food, thereby forming nitrosamines (see below). 

Most alkylating agents found in the environment belong to the class of nitrosamines. The class of nitrosamides is frequently used in basic research or as drugs for the treatment of various tumor entities. In addition, compounds such as alkylsulfonates and nitrogen mustards also act via DNA alkylation. 

### 1.1. Nitrosamines (N-Nitrosamines)

Nitrosamines represent compounds with the chemical structure R^1^R^2^N−N=O (R = aryl or alkyl group). They are pro-carcinogens, which require metabolic activation to form alkylating agents. Nitrosamines undergo enzymatic α-hydroxylation by CYP450 monooxygenases to form dealkylated primary nitrosamines, which further decompose to diazonium ions. Rearrangement and subsequent elimination of nitrogen result in the formation of carbenium ions, the final DNA alkylating species (for details on nitrosamines chemistry, see [11]). Around 300 structurally different nitrosamines are known [12], whereof more than 20 were assessed by the International Agency for Research on Cancer (IARC) according to their carcinogenicity in humans [13]. Nitrosamines are ubiquitously present in the environment (water, air, food) and arise, for example, in tobacco smoke and during industrial processes. Important nitrosamines found in food, personal care products, and drugs are listed in Table 1. 

#### 1.1.1. *N*-Nitrosamines as Contaminants in Food 

**Figure 2:**
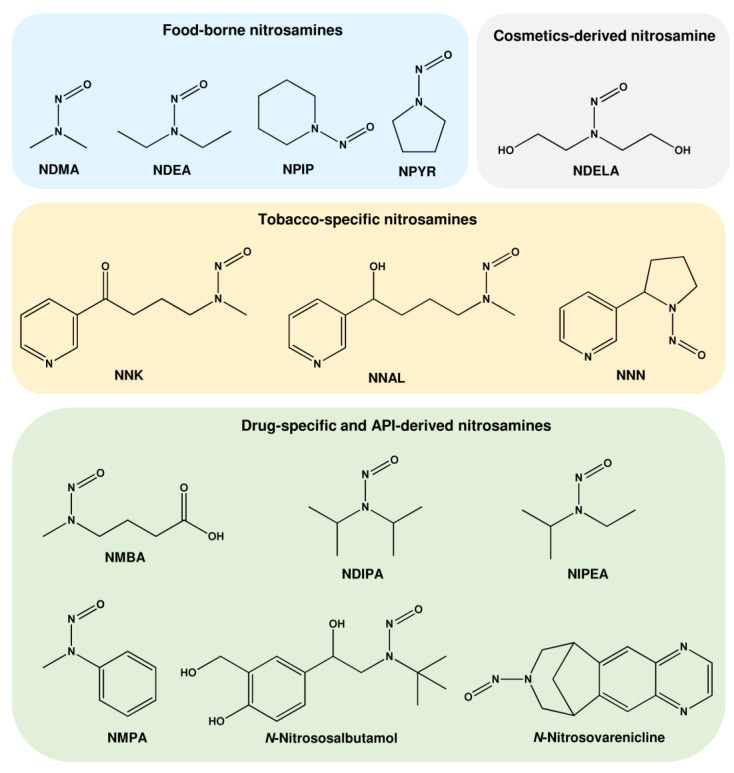
Chemical structure of important nitrosamines found in food, cosmetics, tobacco smoke, and drugs. NDMA: *N*-nitrosodimethylamine; NDEA: *N*-nitrosodiethylamine; NPIP: *N*-nitrosopiperidine; NPYR: *N-*nitrosopyrrolidine; NDELA: *N*-nitrosodiethanolamine; NNK: nicotine-derived nitrosamine ketone; NNAL: nicotine-derived nitrosamine alcohol; NNN: *N*’-nitrosonornicotine; NMBA: *N*-nitroso-*N*-methyl-4-aminobutanoic acid; NDIPA: *N*-nitrosodiisopropylamine; NIPEA: *N*-nitrosoethylisopropylamine; NMPA: *N*-nitrosomethylphenylamine.

The first nitrosamine identified in the environment was *N*-nitrosodimethylamine (NDMA), representing the most prevalent *N*-nitroso compound (NOC) in the diet [14]. Nitrosamines were detected in smoked fish, bacon, sausages, and cheese, but also in beverages such as beer and drinking water [14]. Apart from NDMA, several other nitrosamines were detected in food (Table 1 and Figure 2), including *N*-nitrosodiethylamine (NDEA), *N*-nitrosopiperazine (NPIP), and *N*-nitrosopyrrolidine (NPYR) [15]. The curing process, the temperature, and the amounts of secondary amines are the major factors that influence nitrosamine formation in food [16]. Furthermore, NOCs can be generated endogenously in the gastrointestinal tract, particularly in the stomach and the large intestine [17]. The intake of dietary heme or heme-containing red meat was shown to increase endogenous NOC formation in both rodent models and human volunteers [18,19,20,21]. NOC levels were determined in feces as apparent total nitroso compounds (ATNCs), which include *N*-nitrosamines, S-nitrosothiols, and nitrosyl-iron as major constituents [19,21]. 

**Table 1:** Important nitrosamines, formed DNA alkylation adducts, and their sources.

Nitrosamines	Abbreviation	Major DNA Alkylation Adducts	Sources
*N*-nitrosodimethylamine	NDMA	N7-MeG, N3-MeA *O*^6^-MeG, *O*^2^-MeT, *O*^4^-MeT	Food, drugs, tobacco smoke
*N*-nitrosodiethylamine	NDEA	N7-EtG, N3-EtA, *O*^6^-EtG, *O*^2^-EtT, *O*^4^-EtT	Food, drugs
*N-*nitrosopiperidine	NPIP	7-(2-oxopropyl)-N1,N^2^-etheno-G, N^2^-(3,4,5,6-tetrahydro-2H-pyran-2-yl)-2‘-G	Food
*N-*nitrosopyrrolidine	NPYR	N7,8-ButanoG, N7-(4-Oxobutyl)-G, *O*^4^-(4-OH-Butyl)-T, and others	Food
*N*-nitrosodiethanolamine	NDELA	*O*^6^-OHEtG and others; glyoxal adducts	Cosmetics
*N*-nitroso-*N*-methyl-4-aminobutanoic acid	NMBA	unknown	Drugs
*N*-nitrosodiisopropylamine	NDIPA	unknown	Drugs
*N*-nitrosoethylisopropylamine	NEIPA	unknown	Drugs
*N*-nitrosomethylphenylamine	NMPA	unknown	Drugs
*N*-nitrosovarenicline	-	unknown	Drugs
*N*-nitrososalbutamol	-	unknown	Drugs
4-(methylnitrosamino)-1-(3-pyridyl)-1-butanone; (nicotine-derived nitrosamine ketone)	NNK	N7-MeG, N3-MeA, N3-MeG, *O*^6^-MeG, *O*^4^-MeG, *O*^6^-pobG	Tobacco smoke
4-(methylnitrosamino)-1-(3-pyridyl)-1-butanol; (nicotine-derived nitrosamine alcohol)	NNAL	N7-MeG, N3-MeA, N3-MeG, *O*^6^-MeG, *O*^4^-MeG, *O*^6^-pobG	Tobacco smoke
*N*‘-nitrosonornicotine	NNN	*O*^6^-pobG	Tobacco smoke
*N’*-nitrosoanabasine	NAB	unknown	Tobacco smoke
*N’*-nitrosoanatabine	NAT	unknown	Tobacco smoke

**Figure 3:**
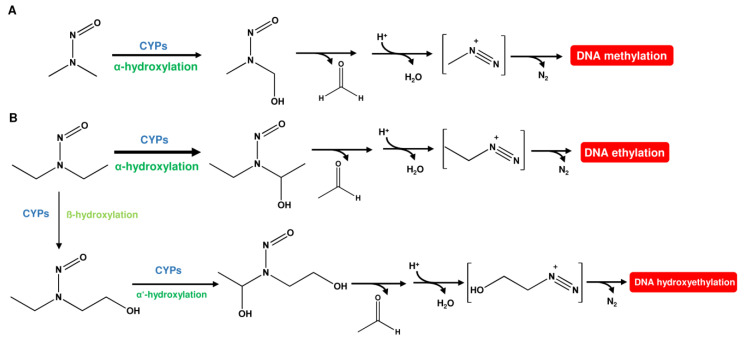
Metabolic activation pathways for NDMA and NDEA. (**A**) *N*-nitrosodimethylamine (NDMA) undergoes hydroxylation by CPYs to form *N*-nitroso(hydroxymethyl)methylamine. This phase I metabolite rearranges under the release of formaldehyde and water to an unstable methyldiazonium ion, which then methylates the DNA. (**B**) *N*-nitrosodiethylamine (NDEA) is primarily hydroxylated at the α-carbon by CYPs. The hydroxylated metabolite then rearranges under the release of acetaldehyde and water to an unstable ethyldiazonium ion, which causes DNA ethylation. NDEA can also undergo ß-hydroxylation as a minor pathway, resulting in the formation of an unstable hydroxyethyldiazonium ion that can hydroxyethylate the DNA.

As mentioned above, *N*-nitrosamines have to undergo metabolic activation to cause DNA damage. NDMA is primarily metabolized by CYP2E1, but also by CYP2A6, via α-hydroxylation [22]. This gives rise to methyldiazonium-ions that react with DNA under the formation of N7-methylguanine (N7-MeG), N3-methyladenine (N3-MeA), and *O*^6^-methylguanine (*O*^6^-MeG) as the most abundant lesions (Figure 3A) [23]. *O*^2^-methylthymine (*O*^2^-MeT) and *O*^4^-Methylthymine (*O*^4^-Me) were only detected at very low levels [23]. NDEA was shown to be activated mainly by CYP2A6 via α-hydroxylation [24], which leads to the formation of the respective ethylated DNA bases, i.e., N7-EtG, N3-EtA, *O*^6^-EtG, *O*^2^-EtT, and *O*^4^-EtT, as prevailing adducts [25]. As a minor pathway, ß-hydroxylation of NDEA can occur (Figure 3B). This gives rise to a 2-hydroxyethyldiazonium ion and DNA adducts such as N7-HOEtG, which is, however, found only in trace amounts [26]. It is further noteworthy that more than 50% of the ethyl adducts were detected at the hydrogen-linked phosphotriester oxygen [25]. 

The metabolic activation and DNA adduct formation of NPIP and NPYR as well as of other food-relevant nitrosamines have been recently reviewed elsewhere [27]. 

#### 1.1.2. *N*-Nitrosamines as Impurities in Cosmetics

*N*-nitrosodiethanolamine (NDELA) was found as the predominant nitrosamine impurity in cosmetics [28] and was reported to be a substrate for CYP2E1-mediated toxification [29]. The metabolism of NDELA can occur via both α- and ß-hydroxylation pathways. The ß-hydroxylation of NDELA results in the formation of *N*-nitroso-2-hydroxymorpholine, which subsequently gives rise to glyoxal, whereas α-hydroxylation yields 2-hydroxyethyldiazonium ions [30]. Therefore, both hydroxyethyl and glyoxal DNA adducts are formed [31]. 

#### 1.1.3. *N*-Nitrosamines Formed by Smoking

Another source of *N*-nitrosamines is cigarette smoke, which contains tobacco-specific nitrosamines [32]. Among them, the nitrosation products of nicotine, namely *N*-nitrosonornicotine (NNN) and nicotine-derived nitrosamine ketone (NNK), have been shown to contribute to cancer risk [33,34,35]. Additionally, nitrosation of anabasine and anatabine generates *N*-nitrosoanabasine (NAB) and *N*-nitrosoanatabine (NAT). Reduction of NNK and NNA forms nicotine-derived nitrosamine alcohol (NNAL) and iso-NNAL, whereas oxidation of NNA generates iso-NNAC. NNK undergoes metabolic activation by hepatic CYP2A6 and respiratory CYP2A13, with the latter being much more efficient [36,37]. NNAL was also reported to be metabolized by lung CYP2A13 [38]. Metabolism of NNK produces either carbenium ions or pyridyloxobutylating (pob)-agents, which lead to the formation of DNA methylation adducts (N7-MeG, N3-MeA, N3-MeG, *O*^6^-MeG, *O*^4^-MeG) and various pob-adducts, with O(6)-[4-oxo-4-(3-pyridyl)butyl]guanine (*O*^6^-pobG) being among them [39]. Thus, *O*^6^-pobG represents the second frequent pyridyloxobutylation product after the corresponding N7 alkylguanine adduct 7-[4-(3-pyridyl)-4-oxobut-1-yl]-guanine (N7-pobG) in NNK-exposed rats [40]. NNN was shown to be metabolized by both CYP2A6 and CYP3A4 [41]. In opposition to NNK, NNN only induces pob-adducts, with *O*^6^-pobG being among them [42,43]. Both DNA methylation and DNA pyridyloxobutylation are important events in NNK- and NNN-induced carcinogenesis in the rat [44], whereas in the mouse lung, DNA methylation seems to be of higher relevance [45]. For further reading on the metabolism and DNA adduct formation of tobacco-specific nitrosamines, we recommend a recently published comprehensive review [39].

#### 1.1.4. *N*-Nitrosamines as Impurities in Drugs

A couple of years ago, nitrosamines were detected in various batches of sartanes [46], which are angiotensine receptor blockers and frequently prescribed antihypertensive drugs. The sartanes (valsartan and others) contained up to 20 µg of NDMA per tablet [47], which raised a major concern, leading to one of the largest drug recalls across Europe and the US. Metformin, a drug used for the therapy of type 2 diabetes, was shown to contain NDMA in late 2019 [48]. A detailed analysis of more than 1000 samples consisting of metformin active pharmaceutical ingredient (API) and drug products revealed that roughly 18% of all samples exceeded the limit of 32 ppb of NDMA, which is based on the acceptable intake (AI) of NDMA (96 ng) multiplied by the maximum daily dose of the API [49]. The AI is defined according to the ICH guideline M7(R1) for genotoxic and potentially carcinogenic impurities in pharmaceuticals and represents the daily, lifelong intake level corresponding to a theoretical cancer risk of 10^−5^ [50]. Interestingly, most of the API samples had no NDMA impurity, while a substantial amount of the finished dosage form was contaminated with NDMA at levels above the AI. Nitrosamines were also found in other drugs including ranitidine, a histamine receptor antagonist, and the antibiotic rifampicin [51]. In addition to short nitrosamines such as NDMA and NDEA, more complex nitrosamines have been identified in APIs and their products, e.g., *N*-nitroso-*N*-methyl-4-aminobutanoic acid (NMBA), *N*-nitrosoethylisopropylamine (NEIPA or NIPEA), and *N*-nitrosomethylphenylamine (NMPA) [52]. Furthermore, nitrosamines derived from the API were detected. Varenicline, a partial nicotinic acetylcholine receptor agonist and a drug used as an aid to smoking cessation, contained the impurity *N*-nitroso-varenicline and was, thus, recalled [53]. Very recently, the drug Ventolin with the API salbutamol was recalled due to the impurity *N*-nitrososalbutamol in three batches [54]. Whether API-derived nitrosamines also occur in other drugs is currently a matter of intensive research. 

### 1.2. Nitrosamides (N-nitrosamides)

**Figure 4:**
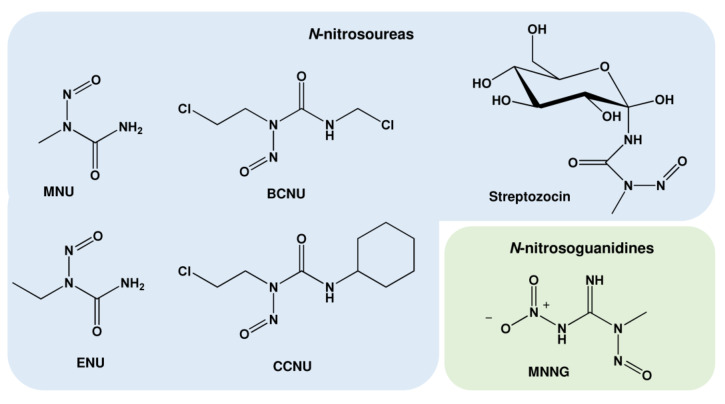
Chemical structure of important nitrosamides. MNU: N-methyl-N-nitrosourea; ENU: N-ethyl-N-nitrosourea; BCNU: 1,3-Bis(2-chloroethyl)-1-nitrosourea; CCNU: 1-(2-chloroethyl)-3-cyclohexyl-1-nitrosourea; MNNG: N-methyl-N’-nitro-N-nitrosoguanidine.

Nitrosamides represent compounds with the chemical structure R^1^C(=X)N(-R^2^)-N=O (R: hydrogen atoms or organic residues) and can be divided into the *N*-nitrosamides (R^1^-C(=O)N(-R^2^)-N=O) and the derivates *N*-nitrosoureas (R^1^R^2^N(=O)N(-R^3^)-N=O), *N*-nitrosoguanidines (R^1^R^2^N(=NH)N(-R^3^)–N=O), and *N*-nitrosocarbamates (R^1^-O-C(=O)N(–R^2^)–N=O). Opposite to nitrosamines, nitrosamides do not require metabolic activation, but decompose spontaneously in aqueous medium, forming diazonium ions and finally carbenium ions as alkylating species. The most relevant class among the nitrosamides are the *N*-nitrosoureas, which comprise highly mutagenic compounds used in basic research as well as multiple compounds used in anticancer therapy (Table 2 and Figure 4). 

*N*-Methyl-*N*-nitrosourea (MNU) and its glucose derivate streptozotocin (*N*-(methylnitrosocarbamoyl)-α-D-glucosamine) represent the first generation of methylating anticancer drugs, forming N7-MeG, N3-MeA, N3-MeG, and *O*^6^-MeG [55]. Chloroethylating agents such as carmustine and lomustine are used as anticancer drugs for the treatment of glioblastoma, astrocytoma, malignant melanoma, gastrointestinal and pancreatic cancer, and Hodgkin’s and non-Hodgkin’s lymphoma [56]. These drugs chloroethylate, among others, the *O*^6^-position of guanine, forming *O*^6^-chloroethylguanine (*O*^6^-ClEtG). This adduct is unstable and undergoes intramolecular rearrangement, forming the N1,*O*^6^-ethenoguanine adduct and subsequently a N1-guanine-N3-cytosine interstrand DNA crosslink [57]. Apart from the mentioned *N*-nitrosoureas, MNNG (N-Methyl-N’-nitro-N-nitrosoguanidine) was used as an S_N_1-methylating drug in multiple studies.

### 1.3. Further Alkylating Agents

**Figure 5:**
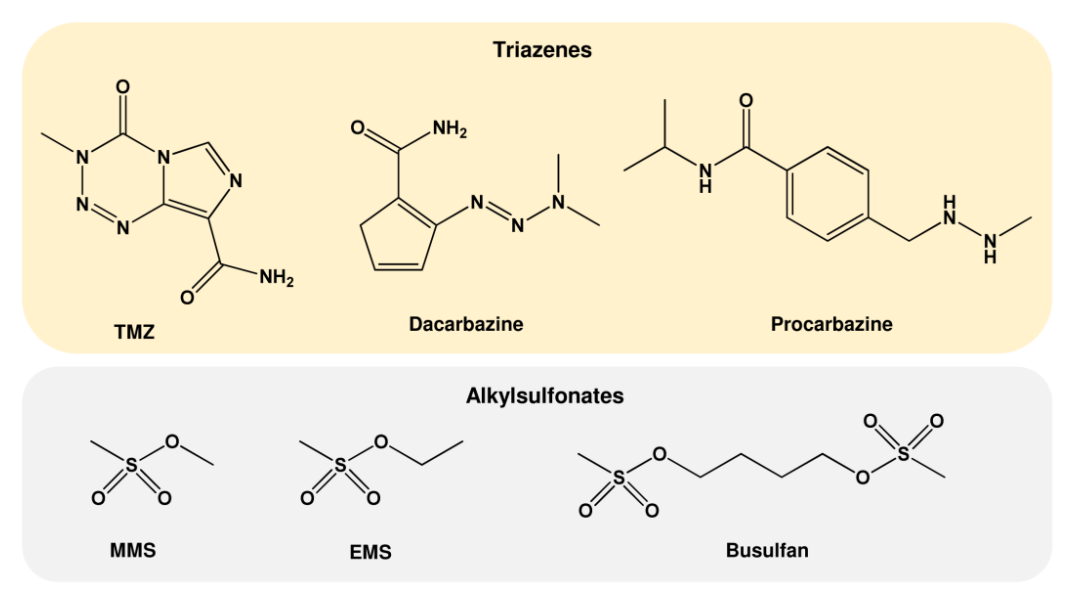
Chemical structure of further alkylating agents. TMZ: temozolomide; MMS: methyl methanesulfonate; EMS: ethyl methanesulfonate.

As mentioned above, MNU was initially used as an anticancer drug. However, due to its unstable nature, it was replaced by the newly designed compounds procarbazine (PCB, PCZ, N-Methylhydrazine, Natulan^®^, Matulane^®^) and dacarbazine (DIC, Imidazole carboxamide, dimethyl-triazeno-imidazole-carboxamide, DTIC^®^-Dome), which generate similar reactive alkylating species (Table 3 and Figure 5) [55]. However, metabolic activation by cytochrome P450 is necessary, which might be an obstacle for anticancer therapy. The latest-generation drug is temozolomide (TMZ, Temodal^®^, Temodar^®^), which does not require metabolic activation and is used in the clinic, preferentially for the treatment of malignant gliomas [58]. TMZ decomposes spontaneously into methyltriazenoimidazole carboxamide (MITC), finally giving rise to methyl carbenium ions [59] similar to those generated by NDMA. Additional alkylating agents not belonging to the group of nitrosamides and nitrosamines are alkylsulfonates such as ethyl methanesulfonate (EMS), methyl methanesulfonate (MMS), and Busulfan, the methanesulfonate diester of 1,4-butanediol. These alkylsulfonates act in an S_N_2 reaction and, therefore, produce mainly N7-MeG and N3-MeA [60].

**Table 3:** Further alkylating agents, DNA alkylation adducts, and their sources.

Further Alkylating Agents	Abbreviations	Major DNA Alkylation Adducts	Sources
**Triazenes**			
N-Isopropyl-4-(2-methylhydrazinomethyl)benzamid	Procarbazine	N7-MeG, N3-MeA N3-MeG, *O*^6^-MeG	Anticancer drug
5-(3,3-Dimethyl-1-triazenyl)imidazol-4-carboxamid	Dacarbazine	N7-MeG, N3-MeA N3-MeG, *O*^6^-MeG	Anticancer drug
4-Methyl-5-oxo-2,3,4,6,8-pentazabicyclo [4.3.0]nona-2,7,9-trien-9-carboxamid	TMZ	N7-MeG, N3-MeA N3-MeG, *O*^6^-MeG	Anticancer drug
**Alkylsulfonates** Ethyl methanesulfonate Methyl methanesulfonate 4-methylsulfonyloxybutyl methanesulfonate	EMS MMS Busulfan	N7-MeG, N3-MeA N7-EtG, N3-EtA N7-MeG, N3-MeA	Basic research Basic research Anticancer drug

## 2. Repair of DNA Alkylation Damage

**Figure 6:**
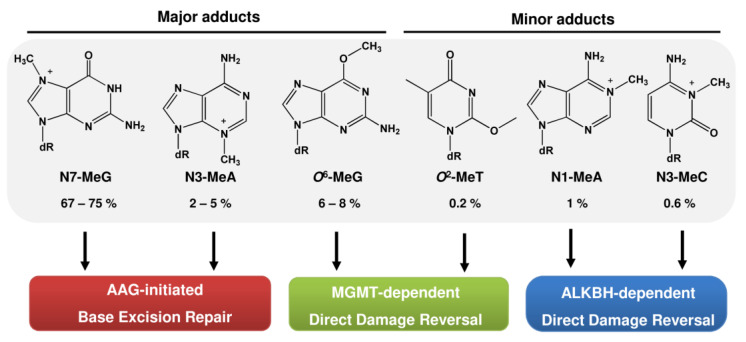
DNA methylation adducts and involved DNA repair pathways. Given is the relative percentage of the total methylated DNA adducts in rat liver following exposure to NDMA for 2 h [23]. AAG: alkyladenine glycosylase; MGMT: *O*^6^-methylguanine-DNA methyltransferase; ALKBH: AlkB homolog.

The different DNA adducts produced by alkylating agents can be repaired by multiple DNA repair mechanisms (Figure 6). Generally, straight-chain alkyl lesions at the N3-A, N3-G, and N7-G position are removed by base excision repair (BER) initiated by the alkyladenine glycosylase (AAG). The AlkB homolog (ALKBH) demethylases directly revert N1-MeA, N1-MeG, N3-MeT, and N3-MeC, while the *O*^6^-methylguanine-DNA methyltransferase MGMT removes alkyl adducts from the *O*^4^-G and *O*^6^-G position. Among them, MGMT can also remove *O*^6^-ClEtG. However, after the formation of crosslinks, MGMT is not effective and the crosslink has to be resolved by the interstrand crosslink repair mechanism. Besides these mechanisms, the nucleotide excision repair pathway (NER) is involved in the repair of bulky alky adducts, and replication-blocking DNA alkylation lesions can be bypassed by translesion synthesis (TLS).

### 2.1. Repair by MGMT

Among the adducts induced by alkylating compounds, alkylations at the *O*^6^-position and *O*^4^-position are removed by the DNA repair protein MGMT. The MGMT gene is located at chromosomal position 10q26.3 and encodes an mRNA of 866 nucleotides and a protein containing 207 amino acids with a molecular weight of 24 kDa [61,62]. MGMT can remove methyl groups from *O*^4^-MeG and *O*^6^-MeG, but not from methylphosphotriesters [63,64]. However, the repair of *O*^6^-MeG is between 10^5^ and 10^3^ times faster than that of *O*^4^-MeG [65]. Besides methyl adducts, longer alkyl adducts can also be repaired by MGMT, such as ethyl-, n-propyl-, n-butyl-, 2-chloroethyl-, 2-hydroxyethyl-, iso-propyl-, and iso-butyl adducts. However, in this case, the repair efficiency decreases with increasing size of the alkyl group [66,67]. Importantly, *O*^6^-pobG can also be repaired by MGMT [68,69,70,71] and, if not repaired, induces G→A and G→T mutations [72].

Although being only a minor alkylation product with less than 8% of total alkylations, *O*^6^-MeG represents the most carcinogenic lesion. Thus, it was shown that the neurotropic carcinogenic activity of MNU depends on the lack of repair of *O*^6^-MeG [73]. In addition, transgenic mice overexpressing MGMT in their skin are protected against tumor formation upon exposure to MNU and nimustine (ACNU) [74,75,76]. Data obtained in different mouse models also revealed that MGMT protects against methylation-induced liver cancer [77], lung cancer [78,79], thymic lymphomas [80,81], and colon cancer [82,83]. 

A landmark study demonstrated that MGMT causes a threshold in NOC-induced colon cancer formation at low methylation dose levels [84]. Wild-type DNA-repair-proficient mice showed a non-linear tumor formation, whereas MGMT-deficient mice developed tumors in a linear, dose-dependent manner [84]. Intriguingly, these findings correlated very well with the NOC-induced *O*^6^-MeG levels and γH2AX formation in colorectal tissue [84,85]. Transgenic mice with high expression levels of the bacterial MGMT homolog Ada showed decreased liver tumor formation after treatment with the nitrosamines NDMA and NDEA [77]. Consistent with this finding, MGMT knockout mice revealed a higher frequency of GC → AT transition mutations in the liver and lung upon treatment with the tobacco-specific nitrosamine NNK [86]. This correlated very well with increased levels of *O*^6^-MeG and *O*^6^-pobG in both organs of MGMT-deficient mice after NNK treatment [86].

Moreover, *O*^6^-alkylating agents are also highly toxic. Thus, repair by MGMT almost completely prevents cell killing at lower concentrations. In line with this, MGMT confers resistance to methylating and chloroethylating anticancer drugs during anticancer therapy in humans [87,88,89]. At high concentrations, other repair pathways, e.g., base excision repair, may become saturated and the N-alkylations then mediate cytotoxicity. 

**Figure 7:**
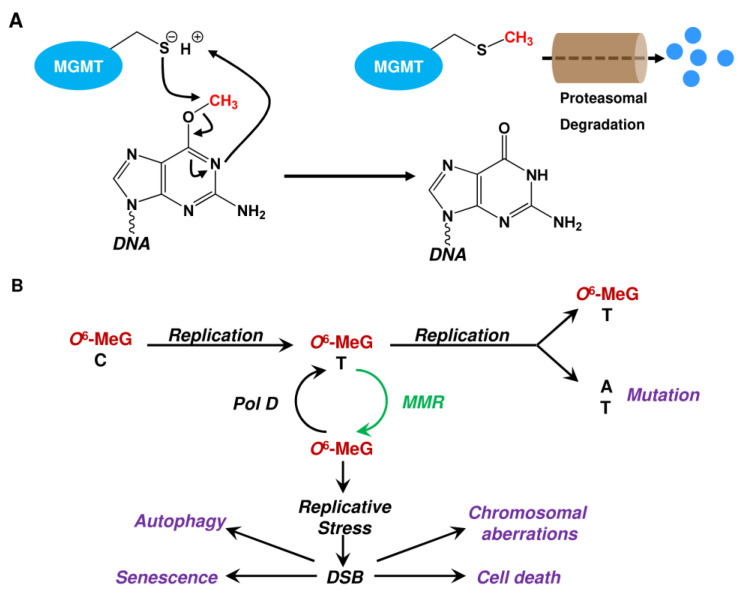
(**A**) Repair of *O*^6^-methylguanine adducts by MGMT. (**B**) Biological consequences of unrepaired *O*^6^-methylguanine (*O*^6^-MeG). Pol D: polymerase delta; MMR: mismatch repair; DSB: DNA double-strand breaks.

Mechanistically, MGMT acts by direct damage reversal. The alkyl group is transferred from the *O*^6^-group of guanine onto a cysteine residue (Cys145) in the active center of MGMT in a one-step reaction, thereby restoring guanine [63,64]. Alkylated MGMT is thereafter ubiquitinated and degraded by the proteasome [90] (Figure 7A). Therefore, the repair capacity of *O*^6^-alkylation damage is determined by the cellular MGMT level. However, as *O*^6^-MeG does not interfere with replication, it is not toxic by itself and replication is required for toxicity [91]. If not repaired by MGMT, *O*^6^-MeG mispairs with thymine (T) during the first round of replication [92] (Figure 7B).

Within the second round of replication, the generated *O*^6^-MeG-T mispair is converted into an A→T transversion in one of the daughter cells, whereas the *O*^6^-MeG-T mispair is retained in the second. For toxicity, the *O*^6^-MeG-T mispair has to be processed by the mismatch repair (MMR) system [93]. However, MMR acts on the newly synthesized strand, where it removes the thymine, leaving the *O*^6^-MeG behind. As consequence, thymine is re-incorporated opposite *O*^6^-MeG at high frequency. Subsequent additional rounds of repair by MMR and reinsertion of T destabilizes the DNA in a futile repair cycle. Destabilization is caused by the large size of the DNA stretches removed by MMR [94]. The generated single-stranded DNA is quite unstable and can break, e.g., when encountering the replication machinery, leading to the formation of toxic DNA double-strand breaks (DSBs) [95]. We should note that it has been estimated that only ~1% of the endogenously formed SSBs can be converted into DSBs [96]. Concerning *O*^6^-MeG, it has been shown that treatment of A172 cells with 20 μM TMZ induces 14,000 *O*^6^-MeG adducts, which are converted into 32 DSBs determined as γH2AX foci, representing a conversion rate of 0.23% [97]. If not repaired by homologous recombination (HR) [98] in the post-treatment cell cycle [99], DSBs trigger downstream pathways, such as cell death and senescence (Figure 7B). As already mentioned, *O*^6^-MeG is not highly toxic, which is also observed in glioma cells exposed to TMZ. In this case, unrepaired *O*^6^-MeG and the subsequent DSBs trigger mostly senescence and little cell death [100,101].

*O*^6^-ClG adducts can also be repaired by MGMT. If not repaired, *O*^6^-ClG undergoes intramolecular rearrangement, forming the N1-*O*^6^-ethenoguanine adduct and subsequently a N1-guanine-N3-cytosine interstrand DNA crosslink [57]. If not removed, these interstrand crosslinks block DNA replication and lead to DSBs, with subsequent cell death induction [102].

Strong differences in MGMT activity and expression have been observed in humans [103]. The differential expression and activity of MGMT are most likely caused by multiple polymorphisms found in the MGMT gene and its promoter. However, an association between these polymorphisms and cancer risk has not been proven (for further reading, see [104]). Moreover, the MGMT expression can be regulated epigenetically and transcriptionally. In rodent cells, MGMT expression is regulated at the transcriptional level and its expression can be increased upon exposure of cells to alkylating agents. Thus, the basal MGMT expression depends on the transcription factors p53 and SP1 [105,106,107,108]. In this case, p53 has been shown to bind and sequester SP1 by preventing its binding to the MGMT promoter [109]. MGMT induction was also reported in rodent cells upon genotoxic stress such as UVC, ionizing radiation, and alkylating agents as well as corticosteroids [110,111,112,113,114,115] and in human HeLaS3 cells upon treatment with different activators of protein kinase C (PKC) such as phorbol-12-myristate-13-acetate (TPA) and 1,2-diacylglycerol (DAG) [115]. In human cells, transcriptional regulation of MGMT is also controlled by SP1 and upregulated by glucocorticoids, but not by genotoxic stress such as TMZ and radiation [116]. In humans, MGMT expression is regulated via epigenetic mechanisms. Thus, methylation of CpG islands localized between -249 and -103 as well as between +107 and +196 within the promoter induces transcriptional silencing [117,118,119,120,121]. As a consequence, the MGMT activity differs between different organs, being highest in the liver and lowest in the brain, myeloid tissue, and hematopoietic stem cells (for review, see [122]) and also between different individuals. Using peripheral blood mononuclear cells (PBMCs), high inter-individual but only moderate intra-individual variations were observed [123]. Concerning MGMT expression during development, it was shown that fetal liver has a lower MGMT activity than the corresponding adult tissue, whereas in most other paired tissues, the activities are in the same range [124]. Moreover, reduced MGMT expression was observed during cytokine-stimulated in vitro maturation of peripheral blood monocytes into dendritic cells [125]. Of note, MGMT promoter methylation with subsequent abrogated expression and activity is frequently observed in different tumors such as brain tumors [126]. Therefore, MGMT is a predictive marker for the effectiveness of methylating anticancer drugs, and clinical trials are underway analyzing the influence of MGMT inhibition on the therapeutic success (for further reading, see [127,128]).

Apart from its epigenetic and transcriptional regulation, MGMT is influenced on the protein and activity level by natural compounds and drugs. On the one hand, antioxidants such as curcumin, cysteine prodrugs such as Oltipraz and N-acetylcystein, and coffee diterpenes were shown to increase MGMT levels and activity in cultured cells and in rat liver [129,130]. On the other hand, the disulfide compounds disulfiram and α-lipoic acid were identified as direct MGMT inhibitors that trigger MGMT depletion in cancer cells [131,132]. Furthermore, the nitric oxide donor S-nitroso-N-acetylpenicillamine was reported to downregulate MGMT expression in glioma [133], while the anti-estrogen tamoxifen was shown to promote MGMT degradation in colorectal cancer cells [134]. Changes in MGMT level and activity have a tremendous impact on the susceptibility of cells, tissues, and organs toward S_N_1 alkylating agents such as nitrosamines and anticancer drugs as pointed out above. 

Overall, MGMT represents the most important pathway in the repair of DNA alkylation damage at the *O*^4^ and *O*^6^ position of guanine, which is typically induced by nitrosamines.

### 2.2. Repair by the ALKBH Family

**Figure 8:**
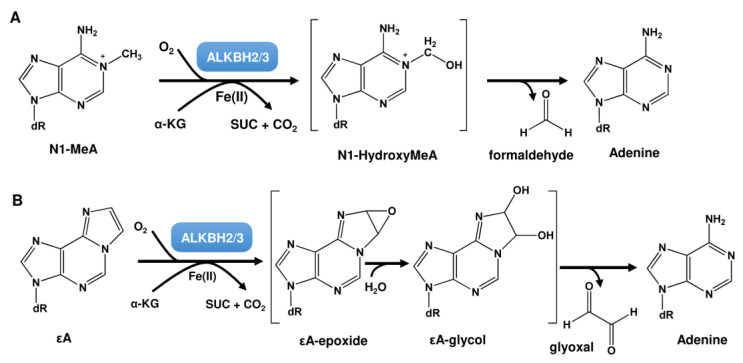
ALKBH-mediated repair of (**A**) N1-MeA and (**B**) εA. α-KG: alpha-ketoglutarate; SUC: succinate; dR: deoxyribose moiety.

Apart from MGMT, a second direct reversal mechanism exists, which targets N-alkyl lesions and consists of the ALKBH demethylase family. These demethylases were first described in *E. coli* as part of the adaptive response [135]. During this response, upregulation of the MGMT-like repair protein Ada and the oxygenase AlkB was described. AlkB was shown to repair DNA alkylation damage such as N1-MeA and N3-MeC in an oxygen, alpha-ketoglutarate (α-KG), and Fe(II)-dependent reaction, by coupling oxidative decarboxylation of α-KG to hydroxylation of methylated DNA bases [136,137] (Figure 8A). N1-MeA and N3-MeC represent replication-blocking lesions. Thus, in AlkB-deficient cells, only ~12% of them are bypassed. Furthermore, N3-MeC is strongly mutagenic, inducing C → T and C → A mutations, whereas N1-MeA caused little mutagenicity [138]. Apart from N1-MeA and N3-MeC, AlkB also repairs N1-MeG and N3-MeT with lower efficiency [138]. Both lesions are also highly mutagenic. N1-MeG induces G→T, G→A, and G→C mutations, whereas N3-MeT induces T→A and T→C mutations. Whereas AlkB preferentially repairs N1-MeA and N3-MeC in single-stranded DNA (ssDNA), N1-MeG and N3-MeT are preferentially repaired in double-stranded DNA (dsDNA) [139]. Finally, exocyclic DNA adducts such as 1,*N*^6^-ethenoadenine (εA) and 3,*N*^4^-ethenocytosine (εC) are also substrates of AlkB [140,141,142].

In human cells, nine AlkB homologs (ALKBH1 to ALKBH8 and FTO) are known [143], but only ALKBH2 and ALKBH3 act as α-KG- and Fe(II)-dependent dioxygenases at N1-MeA and N3-MeC [144,145]. The ALKBH2 gene is localized at chromosome position 14q24.11, harbors 4 exons, contains a coding sequence of 786 bp, and encodes a protein consisting of 261 AS with a molecular weight of 33.1 kDa. ALKBH3 is located at position 11p11.2, harbors 10 exons, contains a coding sequence of 7861 bp, and encodes a protein consisting of 286 AS with a molecular weight of 37.9 kDa. As mentioned above, ALKBH2 and ALKBH3 have also been shown to repair εA [140,146], which is thought to occur via epoxide formation at the etheno bond followed by hydrolysis to a glycol derivative, giving rise to glyoxal and the restored adenine base (Figure 8B) [140]. ALKBH2 was also demonstrated to repair εC [147,148]. However, these etheno-adducts are not typical alkylation products, but rather produced by vinyl chloride or lipid peroxidation. Furthermore, N1-MeG and N3-MeT can be repaired, however at lower rates [138,149,150]. ALKBH2 and ALKBH3 are not only involved in DNA repair, but also in epigenetic regulation. Similar to the α-ketoglutarate (α-KG)/Fe(II)-dependent dioxygenase TET (ten-eleven translocation), ALKBH2 and ALKBH3 can oxidize 5-methylcytosine (5mC) to 5-hydroxymethylcytosine (5hmC), 5-formylcytosine (5fC), and 5-carboxylcytosine (5caC) [151].

Mouse embryonic fibroblasts (MEFs) derived from *Alkbh2* and *Alkbh3* knockout mice were ∼2-fold more sensitive to MMS-induced cytotoxicity as compared to wild-type cells [152]. While the spontaneous mutation frequency was not enhanced in both knockout MEFs, Alkbh2-deficient cells showed an increased mutant frequency after MMS treatment [152]. Another study showed that *Alkbh2*-deficient mice accumulate N1-MeA adducts in the genome [153]. This was not the case in *Alkbh3*-deficient mice, indicating that the reversal of N1-MeA is mainly mediated by ALKBH2 in mammalian cells. However, *Alkbh2/Alkbh3* double-knockout mice are more susceptible than *Alkbh2*-deficient mice to alkylation-induced, inflammation-driven colon carcinogenesis and toxicity, strongly suggesting that both ALKBH2 and ALKBH3 are required for the repair of DNA alkylation damage [154]. This is supported by the finding that ALKBH2 and ALKBH3 show different substrate specificity. Thus, AKBH2 acts preferentially on dsDNA, whereas ALKBH3 acts on ssDNA [144]. In line with this, ALKBH2 but not ALKBH3 can interact with PCNA during replication [155,156]. In opposition to that, ALKBH3 interacts with the ssDNA-binding proteins recombinase A (RecA) and RAD51C [157]. This interaction is supposed to recruit ALKBH3 to alkylation lesions at the 3’-tailed DNA generated during homologous recombination. Novel findings indicate that the recruitment of ALKBH3 to the DNA is associated with transcriptional-coupled repair [158,159]. Thus, upon stalling of the RNA polymerase II, RNF113A ubiquitinates several transcription-associated proteins, leading to recruitment of the ASCC (activating signal co-integrator complex) via the ubiquitin-binding subunit ASCC2 [160]. In a subsequent step, the ASCC helicase subunit ASCC3 unwinds the DNA, thereby generating ssDNA, allowing ALKBH3 to target the alkylation lesion also in the context of dsDNA [161]. Importantly, deficiency in all ASCC components sensitizes cells to alkylating agents [158,159,160,161].

As mentioned above, ALKBH2/3 can revert N1-MeA and N3-MeC lesions. These lesions are not the major lesions induced by clinically used alkylating agents. Especially N1-MeA is thought to be non-cytotoxic because of the efficient repair by ALKBH proteins. Therefore, it is surprising that ALKBH2 has been reported to confer resistance against TMZ in human glioblastoma cells [162]. Moreover, a positive correlation between promoter methylation of ALKBH3 and cellular N3-MeC levels was shown in breast cancer cell lines, suggesting a role in alkylation-based chemotherapy [163]. In glioblastoma, the ALKBH-dependent repair might also be associated with the superior prognosis of IDH1 (Isocitrate dehydrogenase 1) mutant tumors. Mutant IDH1 can convert α-KG into the oncometabolite D-2-hydroxyglutarate, which can directly inhibit ALKBH-dependent repair [164] and sensitizes cells to alkylating agents such as MMS and MNNG [165] and even CCNU [166]. 

Taken together, repair by the ALKBH family plays only a role at minor DNA lesions such as N1-MeA, N3-MeC, N1-MeG, and N3-MeT. 

### 2.3. Repair by Base Excision Repair (BER) Initiated by AAG and Role of PARP-1

**Figure 9:**
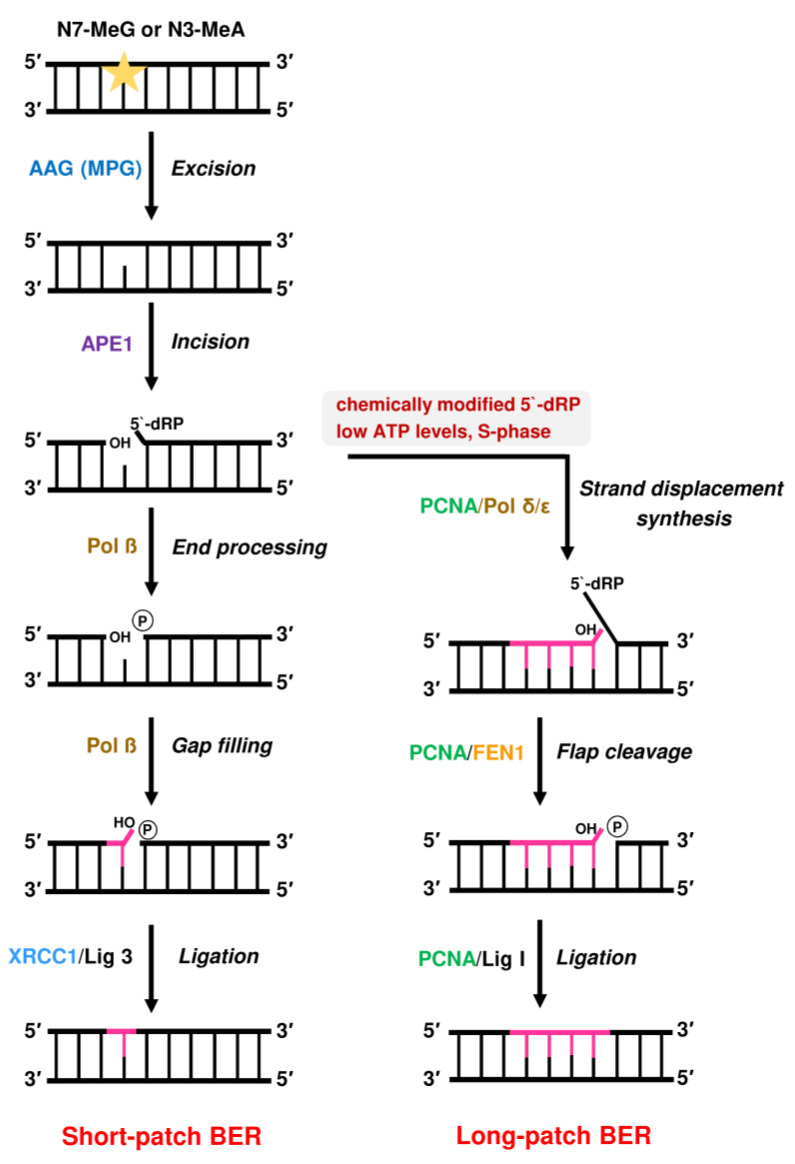
Base excision repair (BER) of DNA alkylation adducts such as N7-MeG or N3-MeA.

BER is a highly conserved pathway, which is responsible for the removal of *N*-methylated DNA adducts, such as N7-MeG and N3-MeA (Figure 9). Apart from that, BER is involved in the repair of oxidative DNA damage, e.g., 8-Oxoguanine, and DNA damage resulting from spontaneous deamination, such as the conversion of cytosine to uracil [167]. The *N*-methylated DNA lesions are detected by the enzyme AAG, which is also called N-methylpurine-DNA glycosylase (MPG) [168,169]. Further substrates of AAG include hypoxanthine (Hx), εA, and N1-MeG [170]. Both εA and N1-MeG are also substrates for ALKBH-mediated direct repair as mentioned above [140,150]. Whether larger N-alkyl adducts, such as N7-EtG, are removed by AAG has not been tested so far. However, it is known that N3-EtA and N7-EtG are prone to undergo spontaneous depurination, resulting in the formation of an apurinic site (AP site) [171]. This process also occurs with N7-MeG adducts, albeit with slower kinetics [172].

AAG null mouse embryonic stem (ES) cells are hypersensitive to alkylation-induced cell killing and chromosomal damage as shown after treatment with MMS and BCNU [173]. In line with that, an increased number of mutations was found in splenic lymphocytes of AAG-deficient mice exposed to MMS [174]. Furthermore, MEFs derived from AAG^-/-^ mice are hypersensitive to the alkylating agent MeOSO_2_(CH_2_)_2_-lexitropsin [168]. This is a methylsulfonate ester covalently attached to N-methylpyrrolecarboxamide dipeptide, which displays DNA minor groove binding and, thus, predominantly induces N3-MeA [175]. Exposure of AAG^-/-^ ES cells to MeOSO_2(_CH_2_)_2_-lexitropsin caused chromosomal aberrations, p53 accumulation, and apoptosis [176]. AAG-deficient ES cells were further reported to show higher initial N3-MeA levels upon MNU treatment and a strongly attenuated repair as compared to wild-type ES cells, highlighting the role of AAG-mediated repair [177]. On the other hand, overexpression of AAG was demonstrated to render cells hypersensitive to DNA alkylation damage by MMS, resulting in increased chromosomal instability [178]. This study provided the first evidence that imbalanced AAG-mediated BER is detrimental to cells. The hypersensitivity phenotype was also observed after mitochondrial overexpression of AAG and MMS treatment [179]. Overexpression of AAG was further reported to increase the sensitivity of cells toward TMZ [180], which is potentiated by the BER inhibitor methoxyamine and Poly(ADP-ribose) polymerase (PARP) inhibitors [181]. 

Increased colon cancer formation was observed in AAG^-/-^ mice initiated with the NOC-related compound azoxymethane (AOM) in combination with the tumor promoter dextran sodium sulfate (DSS) that triggers colitis [82,182]. Interestingly, dose–response studies with AOM revealed a non-linear colon cancer formation in AAG-deficient mice with a threshold similar to that of wild-type animals, but with increased tumor formation at higher AOM doses [84]. A very recent study investigated the role of AAG in liver carcinogenesis induced by NDMA [5], which causes N7-MeG, N3-MeA, and *O*^6^-MeG as the most abundant lesions. As N7-MeG is of minor relevance due to its spontaneous depurination and *O*^6-^MeG is removed by MGMT, the observed effects were primarily attributed to the induced N3-MeA and its replication-blocking properties. The lack of AAG increased mutation rates in liver and promoted liver cancer formation, whereas AAG overexpression resulted in fewer mutations, but more liver tissue damage and lethality [5]. The latter phenotype is very likely due to the fast removal of N3-MeA in mice with AAG overexpression, which leads to an accumulation of SSBs as repair intermediates. These SSBs can then collide with the replication fork, thereby causing replication fork collapse and DSB formation [183]. These findings illustrate the importance of balanced AAG levels to prevent mutagenicity on the one hand and tissue damage on the other hand after exposure to alkylating agents such as nitrosamines. Intriguingly, AAG levels in PBMCs were reported to vary up to 10-fold between human individuals [184,185]. 

On the biochemical level, AAG is a monofunctional enzyme that harbors only a DNA glycosylase activity, whereas bifunctional DNA glycosylases such as OGG1 (8-oxoguanine DNA glycosylase 1) possess an additional AP lyase activity [167]. AAG catalyzes the hydrolysis of the N-glycosidic bond between the damaged DNA base (e.g., N7-MeG or N3-MeA) and the deoxyribose moiety [168,186] (Figure 9). This process may be stimulated by UV-DDB, as shown very recently for the AAG substrates εA and Hx *in vitro* [187]. The release of the damaged base generates an AP site. Subsequently, AP endonuclease (APE1) catalyzes the incision of the phosphodiester backbone at the AP site. This reaction generates a DNA nick with a 5′-deoxyribose-5-phosphate (5′-dRP) and a free 3′-OH group [188]. The 5`dRP moiety is eliminated by DNA polymerase ß (Pol ß) via its intrinsic lyase activity, which produces a 5′-phosphate terminus [189]. BER is then typically completed via the so-called “short-patch” pathway, in which Pol ß catalyzes the incorporation of a new nucleotide using the 3′-OH group as a primer [190]. Finally, the remaining nick is sealed by DNA ligase III in a complex with the scaffold protein X-ray repair cross-complementing protein 1 (XRCC1), which stabilizes DNA ligase III [191,192]. Furthermore, XRCC1 interacts with Pol ß, which was shown to be required for efficient BER [193]. It should be mentioned that DNA ligase I may substitute for DNA ligase III in short-patch BER, which occurs in an XRCC1-independent manner [194]. 

If the 5′-dRP moiety is chemically modified and is, therefore, no substrate for Pol ß, BER proceeds via the “long-patch” pathway. Long-patch BER is also the prevailing pathway during the S-phase of the cell cycle and at low ATP levels [167,195,196]. This pathway involves additional factors including Pol δ and ε, PCNA, flap endonuclease-1 (FEN1), and DNA ligase I [197]. First, Pol δ and ε catalyze strand displacement synthesis with a stretch of 2-13 nucleotides, thereby generating a so-called 5′-flap structure [198]. In the next step, this flap structure is removed by FEN1 [195] and the remaining nick is sealed by DNA ligase I [199].

Furthermore, the nuclear protein PARP-1 is an important factor that accelerates BER, although not being essential for this process [200,201]. PARP-1 is a DNA damage sensor and activated by DNA strand breaks, including SSB intermediates arising during BER [202,203]. Upon activation, PARP-1 catalyzes the synthesis of poly(ADP-ribose) (PAR) with consumption of NAD^+^ [204]. The formed biopolymer is covalently attached to PARP-1 itself and to other acceptor proteins, such as histones, chromatin remodelers, and DNA repair factors [205,206]. This results in direct or indirect modulation of the local chromatin structure, thereby facilitating the BER process [201]. Furthermore, PARP-1 recruits XRCC1 and other repair proteins through their PAR-binding motif, allowing for a non-covalent interaction with high affinity [207,208,209]. PAR binding to XRCC1 is indispensable for XRCC1 function in BER and SSB repair [210]. On the other hand, XRCC1 in its complex with Pol ß and DNA ligase III has recently been shown to curtail excessive PARP-1 activation at formed SSB intermediates, which can result in PARP-1-mediated cytotoxicity [211]. The relevance of PARP-1 for DNA alkylation damage was demonstrated in PARP-1 null mice and MEFs derived thereof, which are hypersensitive towards both MNU and MMS with increased levels of DNA damage [212,213,214]. This was also illustrated in colorectal cancer cells with PARP-1 deficiency, which display increased DNA strand break levels after treatment with TMZ [215]. Loss of PARP-1 in vivo caused increased levels of deletion mutations following exposure to *N*-nitrosobis(2-hydroxypropyl)amine (BHP) [216]. In line with this finding, PARP-1-deficient mice were shown to be susceptible to BHP-induced liver cancer [217]. Furthermore, PARP-1^-/-^ mice exposed to the NOC-related compound AOM develop a higher number of colonic tumors and liver nodules [218]. Colon tumor induction upon AOM/DSS challenge was potentiated in MGMT and PARP-1 double knockout mice [215], demonstrating the important roles of these proteins in the protection against NOC-induced genomic instability and cancer. However, this study further revealed that PARP-1 promotes inflammation-driven tumor progression, highlighting the opposing functions of PARP-1 during tumorigenesis [215]. 

Overactivation of PARP-1 in response to severe DNA damage results in PARP-1-mediated cell death [185,219]. This is associated with NAD^+^ depletion and rapid ATP loss, which is caused by PARP-1-dependent suppression of glycolysis via PARylation of hexokinase I [220,221]. In line with this notion, genetic or pharmacological abrogation of PARP-1 protected against MMS-induced, AAG-dependent tissue damage and neuronal degeneration [185,219]. PARP-1 overactivation can directly trigger cell death via its product PAR, which is called PARthanatos [222]. This PARP-1-dependent cell death pathway is observed particularly in neuronal cells, e.g., following oxidative injury or glutamate excitotoxicity, and is characterized by a PAR-triggered release of apoptosis-inducing factor (AIF) from mitochondria [222,223,224,225]. AIF was shown to translocate to the nucleus, where it interacts with macrophage migration inhibitory factor, resulting in neuronal cell death [226]. More recently, PARP-1 was also demonstrated to play a central role in PARthanatos observed in cancer cell lines following treatment with the alkylating agents MNNG and MMS, which was dependent on the MGMT level [227]. Overall, these studies illustrate the close connection of the DNA repair proteins AAG, MGMT, and PARP-1 in genome protection and cell death.

Taken together, BER is the main DNA repair pathway for DNA alkylation adducts formed at the N-position of nucleotides by both S_N_1 (e.g., nitrosamines and nitrosoureas) and S_N_2 alkylating agents (e.g., MMS or EMS). 

### 2.4. Repair by Nucleotide Excision Repair (NER)

The mechanism of NER has been described in detail in multiple review articles [228,229,230] and is summarized below. NER can repair intrastrand-crosslinks such as UV-light-induced photolesions ((6–4) photoproducts (6-4PPs) and cyclobutene pyrimidine dimers (CPDs)) as well as bulky chemical adducts, induced mostly by natural compounds such as the mycotoxin aflatoxin and the main product of incomplete combustion of organic material, benzo(a)pyrene (B[a]P). Moreover, NER is involved in the repair of interstrand-crosslinks, which can be induced by anticancer agents such as chloronitrosoureas and cisplatin. The repair of interstrand-crosslinks is highly complex and utilizes, besides the NER pathway, homologous recombination, translesion polymerases, and the Fanconia Anemia components (for details on interstrand-crosslink repair, see [231,232]). Factors involved in NER belong to different complementation groups such as xeroderma pigmentosum (XP), Cockayne’s syndrome (CS), and trichothiodystrophy (TTD), which are associated hereditary disorders of the same name.

**Figure 10:**
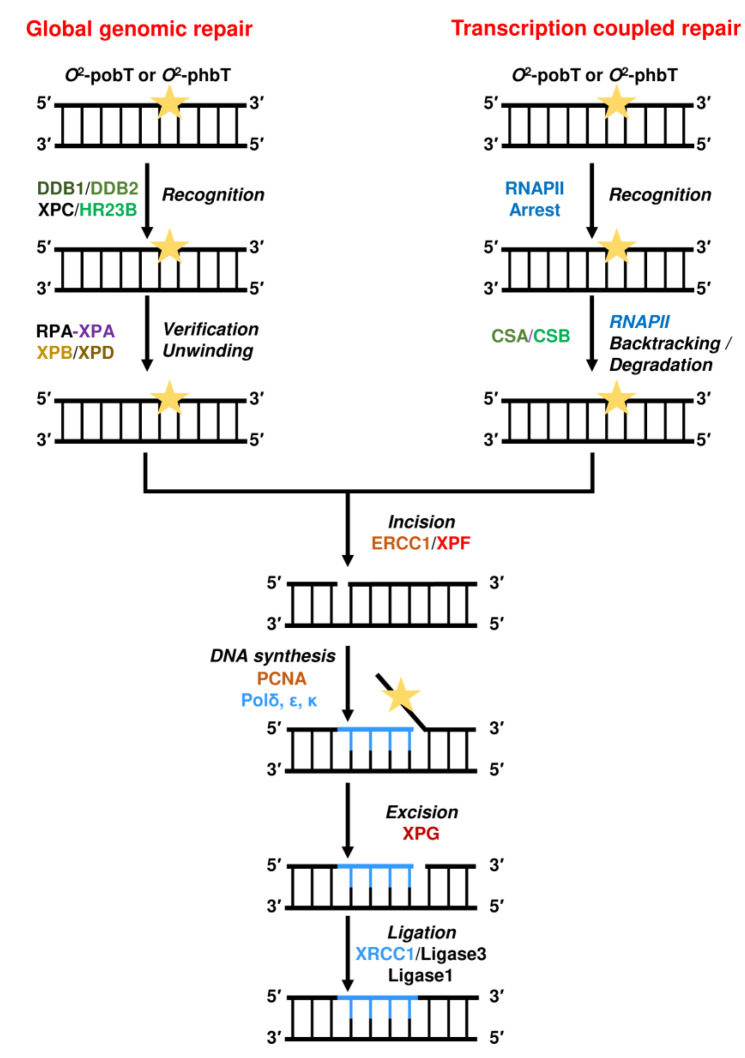
Nucleotide excision repair (NER) of bulky DNA alkylation adducts. Depicted are both global genomic repair and transcription-coupled repair.

NER can be divided in two distinct pathways. The global genomic repair (GGR) removes lesions from the non-transcribed regions of the genome and the non-transcribed strand of transcribed regions, whereas the transcription coupled repair (TCR) removes RNA-polymerase-blocking lesions from the transcribed strand of expressed genes (Figure 10). 

During GGR, lesions strongly distorting the DNA structure are recognized by the XPC–HR23B complex and thereafter verified by the RPA–XPA complex. Lesions that do not strongly distort the DNA helix are recognized by the complex DDB1–DDB2 (XPE). As an example, XPC–HR23B recognizes (6–4)PPs [233], whereas the DDB1–DDB2 complex recognizes CPDs [234]. After recognition of the lesion, the transcription factor TFIIH unwinds the DNA around the lesion [235]. TFIIH consists of two helicase subunits, XPB and XPD, and several accessory components (GTF2H1, GTF2H2, GTF2H3, GTF2H4, CDK7, CCNH, and MNAT1). After unwinding and formation of an open complex, the XPF–ERCC1 complex performs an incision 5′ of the DNA lesion. Thereafter, DNA synthesis by PCNA and the DNA polymerases (δ, ε, and/or κ) leads to the formation of a flap, which is removed by XPG-mediated 3′ incision and the nick is sealed by DNA ligase I, or the Ligase-III-XRCC1 complex [236]. However, if the damage load is too high, DNA synthesis is inhibited and the 3′ incision by XPG does not occur. In this case, XPG can be replaced by EXO1, which further excises the DNA, generating long single-stranded DNA stretches, activating the DNA damage response [237]. 

During TCR, the DNA lesion blocks the RNAPII, leading to assembly of CSA, CSB, and the transcription factor TFIIH at the site of the lesion. CSB uses its DNA translocase activity to remove the RNAPII complex from the lesion [238]. However, it is still unclear whether this is mediated by degradation or backtracking of the RNAPII. Nevertheless, after TFIIH is displaced from the lesion, the lesion becomes accessible and is excised by the exonucleases XPF–ERCC1 and XPG. Again, the DNA polymerases (δ, ε, and/or κ) together with DNA ligase I or the Ligase-III-XRCC1 complex restore the DNA. 

Based on the NER mechanism, it seems obvious that especially bulky adducts might be subject to repair. However, as mentioned above, even bulky adducts such as pyridyloxobutyl (pob) DNA adducts are preferentially repaired by MGMT. Thus, multiple studies have shown that MGMT can remove the pob group from *O*^6^-pobG [239,240,241,242]. However, despite the efficient repair of *O*^6^-pobdG by MGMT, the mutation rate following exposure to NNKOAc was not affected in CHO cells [241]. NNKOAc (4-(acetoxymethylnitrosamino)-1-(3-pyridyl)-1-butanol) is an activated form of NNAL, which is used experimentally because it forms exclusively pob adducts in the presence of cellular esterases. This study indicated that other large adducts, which are not repaired by MGMT and tolerated by translesion synthesis (TLS; see section below), may contribute to mutagenesis.

Besides *O*^6^-pobG, tobacco-specific nitrosamines also generate other pyridylbutylating products and the corresponding pyridylhydroxybutylating (phb) products [240,243,244,245,246]. These are the *O*-adducts *O*^2^-[4-(3-pyridyl)-4-oxobut-1-yl]-cytosine (*O*^2^-pobC), *O*^2^-[4-(3-pyridyl)-4-oxobut-1-yl]-2′-deoxythymidine (*O*^2^-pobT), O^4^-[4-(3-pyridyl)-4-oxobut-1-yl]-thymidine (*O*^4^-pobT), O4-[4-(3-pyridyl)-4-hydroxylbut-1-yl]-thymidine *(O*^4^-phbT), *O*^2^-[4-(3-pyridyl)-4-hydroxylbut-1-yl]-thymidine *(O*^2^-phbT), and O^6^-[4-(3-pyridyl)-4-hydroxylbut-1-yl]-2’-deoxyguanosine *(O*^6^-phbG) and the N-adducts N7-pobG, N7-phbG, *N*^6^-(4-oxo-4-(3-pyridyl)-1-butyl)-2*′*-deoxyadenosine (*N*^6^-pobA), and *N^6^*-(4-hydroxy-4-(3-pyridyl)-1-butyl)-2*′*-deoxyadenosine *(N*^6^-phbA). Furthermore, pyridylbutylating and pyridylhydroxybutylating products on the phosphate backbone were detected [247,248]. 

Using an in vitro NER assay with pyridyloxobutylated plasmid DNA as a substrate, it was shown that nuclear extracts from human lymphoid cell lines deficient in XPA and XPC were less active at repairing pyridyloxobutyl adducts than extracts from normal cells. Moreover, the NER-deficient cells were hypersensitive to NNKOAc [249]. However, as, in the used plasmids, 7-pobG, *O*^2^-pobC, *O*^2^-pobT, and *O*^6^-pobG comprised 31.2%, 22.7%, 25.5%, and 20.6% of the total POB adducts, respectively, an association of these effects with a specific adduct was not possible. Additionally, LC-MS/MS-based measurement of these four pob-adducts was compared in MGMT-deficient, MGMT/BER-deficient, and MGMT/NER (XPD)-deficient CHO cells upon NNKOAc treatment [241]. The results showed a reduced repair of only *O*^2^-pobT in NER-deficient cells. The reduced repair was associated with increased AT→TA transversions, suggesting that these mutations are caused by *O*^2^-pobdT. The differential repair of pob-adducts may also be responsible for differences in the susceptibility to NNK-induced carcinogenesis in different organs of the mouse. Thus, binding of XPA and XPB to pob adducts was increased in liver extracts following NNK treatment, whereas it was decreased in lung extracts [250]. 

Similar results were also observed for pyridylhydroxybutylated adducts. A recent study showed that, among the different PHB adducts induced by NNALOAc (4-(Methylnitrosamino)-1-(3-pyridyl)-1-butanol), NER counteracts the formation of *O*^2^-phbdT and *O*^4^-phbdT, whereas MGMT repairs predominantly *O*^6^-phbdG and, with lesser efficiency, *O*^4^-phbdT [251].

NER also seems to be involved in the repair of smaller adducts. Initial results indicating a removal of *O*^6^-n-butyl-guanine (*O*^6^-BuG) by NER were obtained in bacteria [252,253]. Further experiments in DNA repair-deficient eukaryotic cells also showed that the repair of *O*^6^-BuG (induced by *N*-n-butyl-*N*-nitrosourea (BNU)) was not correlated with MGMT activity, but rather with NER efficiency [254,255,256]. In line with this, XPA-deficient cells showed a decreased bypass efficiency of branched-chain lesions (*O*^6^-iPr-dG and *O*^6^-iBu-dG, *O*^6^-sBu-dG) but not on their straight-chain counterparts (*O*^6^-nPr-dG and *O*^6^-nBu-dG) [257].

Using MGMT or NER-deficient cells, it was shown that *O*^6^-EtG was repaired predominantly by MGMT and more slowly by NER [258]. In opposition to that, *O*^2^-EtG and *O*^4^-EtG were neither repaired by MGMT nor NER. Moreover, MGMT and NER participate in the repair of *O*^6^-carboxymethylguanine (*O*^6^-CMeG) [259,260]. This DNA alkylation adduct was found to be associated with red meat intake [18] and is induced by azaserine as well as *N*-nitrosoglycine [261,262]. Interestingly, a crosstalk between NER and BER on alkylated DNA bases has been observed recently. Here, UV-DDB (DDB1/DDB2 complex) was shown to recognize ϵA and stimulated AAG activity [187]. 

Overall, in contrast to the involvement of MGMT, ALKBH proteins, and BER, only limited data suggest an important role for NER in the repair of and protection against DNA alkylation damage.

### 2.5. Bypass by Translesion Synthesis (TLS)

**Figure 11:**
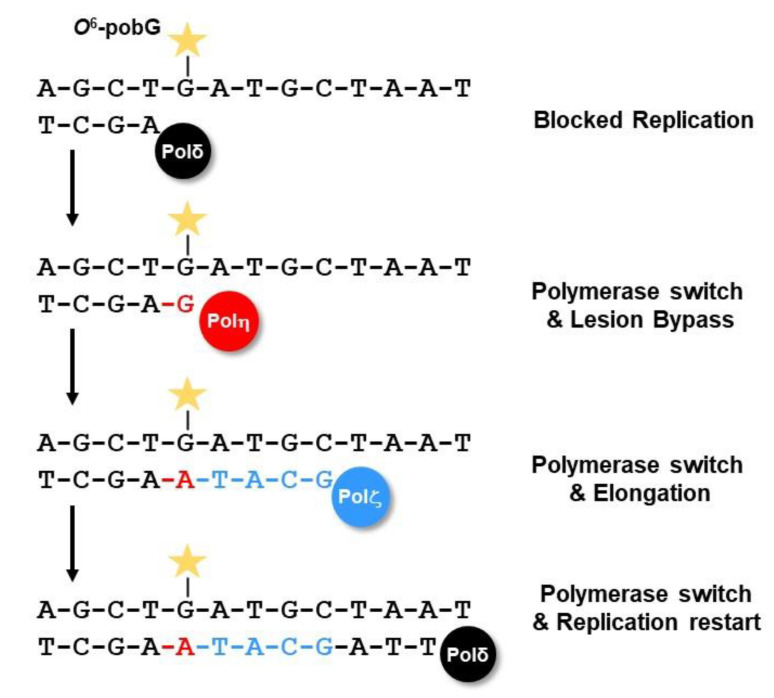
DNA translesion synthesis.

With the exception of small methyl or ethyl adducts, larger and more complex *O*^6^-alkyl-dG lesions strongly block DNA replication. If not repaired, these lesions can cause DSBs and cell death. To avoid this, replication-blocking lesions can be tolerated by a specific bypass mechanism, the translesion synthesis (TLS) (Figure 11). As an example, knockdown of the TLS polymerase eta (Pol η) results in sensitivity to the chloroalkylating anticancer drugs fotemustine and CCNU in melanoma and glioblastoma cells, respectively [263], and knockout of Rev3L leads to hypersensitivity against TMZ and fotemustine [264]. Moreover, RAD18 is required for bypassing TMZ-induced *O*^6^MeG MMR intermediates in the S-phase [265].

TLS utilizes specified polymerases, namely Y-family polymerases (Pol η, Pol ι, Pol κ, and REV1), B-family polymerase (Pol ζ), and A-family polymerases (Pol θ and Pol ν), which can insert nucleotides opposite DNA lesions [266]. In this process, replicative polymerases such as polymerase delta (Pol δ) and polymerase epsilon (Pol ε) are blocked by the lesion. This leads to RAD18/UBE2A-dependent ubiquitination of PCNA at residue K164, and exchange of the replicative polymerase with one of the Y-family polymerases Pol η, Pol ι, or Pol κ. These polymerases can synthesize across the damaged DNA; however, due to their low processivity, they can insert only a few nucleotides [266]. In most cases, only one or two nucleotides are inserted and the Y-family polymerases are thereafter exchanged with the B-family polymerase zeta (Pol ζ, consisting of the subunits Rev3 and Rev7), which (together with Rev1) can extend the newly synthesized DNA. Pol θ can perform both insertion and extension. Finally, the TLS-polymerases are exchanged with the replicative polymerase and normal replication restarts [266]. However, the Y-family polymerases show a weak selectivity and exert no proofreading 3′→5′ exonuclease activity. Therefore, depending on the type of DNA lesion and TLS-polymerase, wrong nucleotides can be inserted and mutations may arise (Figure 11).

#### 2.5.1. Bypass of *O*^6^-alkyl-dG Lesions

Among all *O*^6^-alkyl-dG lesions, only *O*^6^-MeG and *O*^6^-EtG do not strongly block replication and can be bypassed by replicative polymerases [267]. However, it has been shown that also *O*^6^-MeG can slow down Pol α [268] and Pol δ [269] and that Pol η efficiently bypasses *O*^6^-MeG [269]. In this process, all polymerases can insert cytosine or thymine opposite to *O*^6^-MeG, with Pol η acting more accurately, i.e., inserting cytosine. Similar results are obtained for *O*^6^-EtG, which can slow down Pol α and Pol η, leading to error-prone synthesis by both polymerases [270].

In contrast, larger and more complex *O*^6^-alkyl-dG lesions strongly block replicative polymerases. Whereas *O*^6^-MeG did not impede DNA replication, the efficiency of the bypass decreased with the size of the alkyl group [257]. Moreover, the G→A mutation frequency also decreases with the adduct size. In both processes, branched-chain lesions (*O*^6^-iPr-dG and *O*^6^-iBu-dG, *O*^6^-sBu-dG) exerted stronger effects than their straight-chain counterparts (O^6^-nPr-dG and *O*^6^-nBu-dG). Concerning TLS, REV1 is involved in bypassing all of these lesions except *O*^6^-MeG. Whereas straight-chain lesions were mainly bypassed by Pol η and extension was performed by Pol ζ, the branched-chain lesions were predominantly bypassed by Pol ι and Pol κ. 

Another *O*^6^-alkyl-dG lesion bypassed by TLS is *O*^6^-CMeG. Thus, *O*^6^-CmeG can be bypassed by Pol η in an error-prone manner and by Pol κ, which performs an error-free insertion [271]. The subsequent extension step was carried out by Pol η, Pol κ, and Pol ζ.

Data indicate that bulky pyridylbutylating products are also subject to TLS if not repaired by MGMT or NER. Thus, a strong blockage of replicative polymerases was also observed for *O*^6^-pobG, which can be bypassed by Pol η and induces G→A transitions [272]. Furthermore, using recombinant REV1, a one-base incorporation opposite *O*^6^-MeG and *O*^6^-BzG but not *O*^6^-pobG was observed [273]. In this case, REV1 preferentially incorporated dCTP opposite *O*^6^-MeG and *O*^6^-BzG.

#### 2.5.2. Bypass of *O*^2^/*O*^4^-alkyl-dT Lesions

In opposition to *O^6^*-MeG, *O*^2^-MeT and *O*^4^-MeT have been shown to strongly block DNA replication. Moreover, *O*^4^-alkylthymidines have been shown to primarily induce T→C mutations [274], and the exonuclease-free Klenow fragment of *E. coli* DNA polymerase I was shown to incorporate both dATP and dTTP opposite *O*^2^-ethylthymidine [275]. Using different recombinant polymerases, TLS of *O*^2^/*O*^4^-alkyl-dT lesions was further analyzed. In the case of *O*^2^-alkyl-dT lesions, straight-chain lesions (*O*^2^-methylthymidine (*O*^2^-MeT), *O*^2^-ethylthymidine (*O*^2^-EtT), O^2^-propylthymidine (*O*^2^-PrT), *O*^2^-butylthymidine (*O*^2^-BuT)) and corresponding branched-chain lesions (*O*^2^-iPrT, *O*^2^-nBuT, *O*^2^-iBuT, *O*^2^-sBuT) were bypassed by Pol η and, to a lesser degree, Pol κ, but not by Pol ι [276]. Moreover, Pol η was more efficient in incorporating the correct nucleotide opposite *O*^2^-alkyldT lesions with a branched-chain alkyl group than the corresponding lesions with a straight-chain alkyl group [276]. In the case of *O*^4^-alkyldT, all straight-chain lesions besides *O*^4^-MeT and all branched-chain lesions were shown to moderately block DNA replication and to be bypassed by Pol η or Pol ζ, but not by Pol κ or Pol ι [277]. A replication block was also observed for *O*^2^-pobT and O^4^-pobT, which can be bypassed by Pol η and Pol ζ and predominantly induce T→A transversion and T→C transition [278]. Compared to *O*^6^-pobG, this block was more pronounced.

#### 2.5.3. Bypass of N-alkyl-dG Lesions

As mentioned above, among methylated nucleotides, N1-MeA and N3-MeC are the most critical replication-blocking lesions. Therefore, it is obvious that these lesions are also subject to TLS. It has been shown that multiple TLS polymerases are involved in the bypass of N1-MeA [279,280]. (i) Pol ι inserts a nucleotide opposite N1-MeA and Pol θ performs the extension. (ii) Pol η mediates both insertion and extension. (iii) Pol λ inserts a nucleotide opposite N1-MeA and Pol ζ performs the extension. In all cases, TLS is predominantly error-free. Similar results were obtained for N3-MeA. Using 3-deaza-3-methyladenine (3-dMeA), a stable analog of N3-MeA, three different mechanisms were described [281]. (i) Pol ι inserts a nucleotide opposite N3-MeA and Pol κ extends synthesis. (ii) Pol θ can perform both insertion and extension. (iii) Pol ζ extends synthesis by an as-yet-unidentified polymerase. 

Using different recombinant polymerases, Choi and Guengerich analyzed TLS of oligonucleotides containing guanine differentially methylated at the *N*^2^-position *(N*^2^-methylguanine (*N*^2^-MeG), *N*^2^-ethylguanine (*N*^2^-EtG), *N*^2^,*N*^2^-dimethylguanine *(N*^2^,*N*^2^-diMeG), *N*^2^-isobutylguanine (*N*^2^-IbG), *N*^2^-benzylguanine (*N*^2^-BzG), *N*^2^-naphtylguanine (*N*^2^-NaphG), *N*^2^-9-anthracenylguanine (*N*^2^-AnthG), or *N*^2^-6-benzo[a]pyrenylguanine *(N*^2^-BPG)) [272,273,282]. The data indicate that Pol η effectively bypassed *N*^2^-MeG, *N*^2^-EtG *N*^2^-IbG, *N*^2^-BzG, and *N*^2^-NaphG, but not *N*^2^-AnthG and *N*^2^-BPG, whereas Pol δ only bypassed *N*^2^-MeG and *N*^2^-EtG [282]. Pol ι bypassed *N*^2^-MeG as well as *N*^2^-EtG, and partially bypassed *N*^2^-IbG, *N*^2^-BzG, and *N*^2^-NaphG, but was blocked by *N*^2^-AnthG and *N*^2^-BPG [283]. Misinsertion of T increased with the adduct size. Besides Pol η and Pol ι, Pol κ also mediated the bypass of *N*^2^-MeG, *N*^2^-EtG, *N*^2^,*N*^2^-diMeG, *N*^2^-IbG, *N*^2^-BzG, *N*^2^-NaphG, *N*^2^-AnthG, and *N*^2^-BPG. Overall, Pol κ is more efficient than Pol η or Pol ι in incorporating dCTP opposite large *N*^2^-G adducts (*N*^2^-AnthG and *N*^2^-BPG) [284]. Finally, REV1 was shown to perform one base incorporation opposite *N*^2^-MeG, *N*^2^-EtG, *N*^2^,*N*^2^-diMeG, *N*^2^-IbG, *N*^2^-BzG, *N*^2^-NaphG, *N*^2^-AnthG, and *N*^2^-BPG [273]. REV1 preferentially incorporated dCTP opposite G and all of the modified G adducts, with a relatively low misinsertion frequency [273]. Another *N*^2^-adduct is 1,*N*^2^-Etheno(ε)guanine (εG). Whereas Pol δ was completely blocked by this adduct, Pols ι and κ showed similar rates of incorporation of dTTP and dCTP *in vitro*. Pol η was most active, and showed the highest error frequency [285]. Finally, εA has also been shown to be subject to TLS. Thus, in human cells, Pol ι can insert a nucleotide opposite εA and Pol ζ performs the extension [286]. Alternatively, Pol θ can perform both insertion and extension. In both cases, TLS is predominantly error-free.

## 3. Conclusions

Nitrosamines are genotoxic and carcinogenic compounds that occur not only in food, cosmetics, and tobacco smoke, but have also recently been found in drugs as by-products of the API synthesis or even as an API derivative. The DNA adduct formation pathways are well described for food-borne nitrosamines such as NDMA, the cosmetics-related nitrosamine NDELA, as well as tobacco-specific nitrosamines such as NNN and NNK. However, many other nitrosamines, particularly those found in drugs, are insufficiently studied so far with respect to DNA damage induction, or have hitherto even been unknown, thus leaving a data gap. More studies are required to understand the structure–genotoxicity relationship of more complex nitrosamines, such as nitrosamines with larger, branched alkyl residues and API-derived nitrosamines. 

The DNA repair pathways involved in the removal of small DNA alkylation adducts, such as N7-MeG, N3-MeA, or *O*^6^-MeG, are comprehensively understood and mainly involve BER as well as direct damage reversal by MGMT, while ALKBH-mediated repair plays a minor role. The repair of larger *O*^6^-alky adducts caused by tobacco-specific nitrosamines, e.g., *O*^6^-pobG, is also well studied, requiring both MGMT and the NER pathway. In contrast to that, the repair of other DNA alkylation adducts induced, for example, by NDEA or NDELA is hardly characterized so far. Another knowledge gap concerns the repair of adducts generated by complex nitrosamines, such as those found as drug impurities. It is, therefore, necessary to investigate relevant DNA repair pathways in much more detail for those compounds. Such experimental data are eagerly awaited and will be instrumental for in silico approaches to predict the genotoxic (and carcinogenic) potency of poorly characterized or hitherto unknown nitrosamines. Finally, this will be helpful for the risk assessment of nitrosamines to derive AIs for drugs.

## Figures and Tables

**Table 2:** Important nitrosamides, DNA alkylation adducts, and their sources.

*N*-nitrosamides	Abbreviations	Major DNA Alkylation Adducts	Sources
***N-*** **nitrosoureas**			
*N*-methyl-*N*-nitrosourea	MNU	N7-MeG, N3-MeA, N3-MeG, *O*^6^-MeG	Anticancer drug, Basic research
*N*-ethyl-*N*-nitrosourea	ENU	N7-EtG, N3-EtA, N3-EtG, *O*^6^-EtG	Basic research
*N*-(methylnitrosocarbamoyl)-α-D-glucosamine (Streptozocin)		N7-MeG, N3-MeA, N3-MeG, *O*^6^-MeG	Anticancer drug
1,3-Bis(2-chloroethyl)-1-nitrosourea; Carmustine	BCNU	N7-ClEtG, *O*^6^-ClEtG, N1,*O*^6^-EthenoG, G-C and G-G crosslinks	Anticancer drug
1-(2-chloroethyl)-3-cyclohexyl-1-nitrosourea; Lomustine	CCNU	N7-ClEtG, *O*^6^-ClEtG, N1,*O*^6^-EthenoG, G-C and G-G crosslinks	Anticancer drug
***N-*** **nitrosoguanidines**			
*N*-methyl-*N*’-nitro-*N*-nitrosoguanidine	MNNG	N7-MeG, N3-MeA N3-MeG, *O*^6^-MeG	Basic research

## Abbreviations

AAG	alkyladenine glycosylase
ALKBH	AlkB homolog
AP	apurinic/apyrimidinic
APE1	AP endonuclease 1
BER	base excision repair
CPDs	cyclobutane pyrimidine dimers
CS	Cockayne syndrome
CYP450	cytochrome P450
dRP	deoxyribose-5-phosphate
DSB	DNA double-strand break
ERCC1	excision repair cross-complementing 1
EXO1	exonuclease 1
FEN1	flap endonuclease 1
GG-NER	global genome NER
HR	homologous recombination
IR	ionizing radiation
MGMT	*O*^6^-methylguanine-DNA methyltransferase
MMR	mismatch repair
MPG	N-methylpurine-DNA glycosylase (alias AAG)
NER	nucleotide excision repair
OGG1	8-oxoguanine DNA glycosylase
PAR	poly(ADP-ribose)
PARP-1	poly(ADP-ribose) polymerase-1
PCNA	proliferating cellular nuclear antigen
Pol	DNA polymerase
RFC	replication factor C
RNAP	RNA polymerase
RPA	replication protein A
SSB	DNA single-strand break
TC-NER	transcription-coupled NER
TFIIH	transcription factor II H
TLS	translesion synthesis
TTD	trichothiodystrophy
XP	xeroderma pigmentosum
XRCC1	X-ray repair cross-complementing protein 1
6-4PP	pyrimidine-(6,4)-pyrimidone photoproduct
